# Ticagrelor was associated with lower fracture risk than clopidogrel in the dual anti-platelet regimen among patients with acute coronary syndrome treated with percutaneous coronary intervention

**DOI:** 10.1007/s40618-023-02205-1

**Published:** 2023-09-30

**Authors:** D. T. W. Lui, C. H. Wong, A. Ip, A. K. Y. Ng

**Affiliations:** 1 Department of Medicine, School of Clinical Medicine, Li Ka Shing Faculty of Medicine, The University of Hong Kong, Hong Kong SAR, China; 2 Critical Care Unit, School of Clinical Medicine, Li Ka Shing Faculty of Medicine, The University of Hong Kong, Hong Kong SAR, China; 3 Cardiac Medical Unit, Grantham Hospital, Hong Kong SAR, China

**Keywords:** Acute coronary syndrome, Percutaneous coronary intervention, Clopidogrel, Ticagrelor, Osteoporosis, Fractures, Bone, Aspirin

## Abstract

**Purpose:**

Patients with coronary artery disease have increased fracture risks. P2Y12 inhibitors may impact fracture risks. We compared the fracture risks associated with ticagrelor and clopidogrel in dual anti-platelet therapy (DAPT).

**Methods:**

We identified all adults who underwent first-ever percutaneous coronary intervention (PCI) for acute coronary syndrome (ACS) between 2010 and 2017 from a territory-wide PCI registry in Hong Kong. Following 1:1 propensity-score matching for baseline characteristics, patients were followed up till event occurrence, death, or 30 June 2022. Outcomes of interest were major osteoporotic fractures (MOF) identified by validated ICD-9-CM codes. Cox proportional hazards regression was used to compute the hazard ratio (HR) for MOF associated with ticagrelor versus clopidogrel use.

**Results:**

3018 ticagrelor users and 3018 clopidogrel users were identified after propensity-score matching (mean age: 61.4 years; 84.1% men). Upon median follow-up of 6.5 years, 59 ticagrelor users and 119 clopidogrel users sustained MOF (annualized fracture risks: 0.34% and 0.56%, respectively). Ticagrelor use was associated with lower risks of MOF (HR 0.60, 95%CI 0.44–0.83; p = 0.002). Consistent HRs were observed for fractures over vertebrae, hip and upper limbs. Subgroup analyses showed no interaction according to age, sex, presence of diabetes, presence of chronic kidney disease and prior fracture history.

**Conclusion:**

Among adults who underwent first-ever PCI for ACS, ticagrelor use in the DAPT was associated with a lower risk of MOF compared with clopidogrel. Our results support the use of ticagrelor in the DAPT from the perspective of bone health.

**Supplementary Information:**

The online version contains supplementary material available at 10.1007/s40618-023-02205-1.

## Introduction

Osteoporosis is a global pandemic. It is clinically silent until complications occur, manifesting as fragility fractures [1]. Hip and vertebral fractures are the more devastating types of fragility fractures associated with significant morbidities and mortality [2]. Patients with coronary artery disease (CAD) may be at a higher risk of osteoporosis and fragility fractures, potentially mediated through the common pathophysiological mechanism of atherosclerosis [3, 4]. Furthermore, patients with acute coronary syndrome (ACS) tend to be older with multiple comorbidities, increasing the risk of major osteoporotic fractures (MOF). Impairment in activities of daily living after fractures can lead to significant morbidity, creating substantial functional limitations and reducing patients’ quality of life.

Dual anti-platelet therapy (DAPT), consisting of aspirin and a P2Y12 inhibitor, is the standard of care in patients with ACS following percutaneous coronary intervention (PCI) [5]. Inhibition of platelet aggregation by DAPT is deemed essential in reducing the risk of stent thrombosis and ischemic events such as stroke and peripheral artery disease [6]. Recently, novel agents, namely ticagrelor and prasugrel, have emerged as potent P2Y12 inhibitors with better antithrombotic effects than clopidogrel [7, 8]. Observational studies have demonstrated differences in fracture risks associated with different anti-platelet agents [9]. A cohort study by Jorgensen et al. showed that the risk of fracture was increased by approximately 50% among clopidogrel users compared to non-users [10]. Mechanistically, P2Y12 receptors are expressed not only in platelets but also in osteoblasts. Inhibition of P2Y12 receptors by clopidogrel could lower osteoblastic activity and differentiation, reducing new bone formation and thereby increasing fracture risk [11]. On the other hand, ticagrelor, in addition to P2Y12 inhibition, prevents cellular adenosine uptake by the equilibrative nucleoside transporter (ENT)-1, thereby increasing extracellular adenosine levels [12]. This may promote osteoblastic differentiation and bone formation. There are yet observational studies reporting fracture risks associated with ticagrelor use.

Given the higher fracture risks among patients with CAD [13], it is clinically relevant to evaluate whether clopidogrel or ticagrelor is associated with a better fracture risk profile. Based on the observations in the pre-clinical studies, we hypothesize that ticagrelor use is associated with a lower fracture risk than clopidogrel use. Here, we performed a retrospective cohort study comparing the risk of MOF among Chinese patients who underwent PCI and were subsequently treated with clopidogrel versus ticagrelor in the DAPT regimen.

## Methods

### Study population and design

All patients who underwent first-ever PCI between 1 January 2010 and 31 December 2017 in any of all 14 public hospitals that performed PCI in Hong Kong were included in a territory-wide PCI registry [14]. Our current study is a retrospective cohort analysis of all adult patients (aged ≥ 18 years) who underwent PCI for ACS and survived beyond hospital discharge. Patients were excluded from the analysis if they were not prescribed ticagrelor or clopidogrel upon discharge. Patients’ baseline comorbidities, laboratory parameters and concomitant medications were retrieved from the PCI Registry and Clinical Data and Analysis Reporting System (CDARS) [14].

The study was approved by the Institutional Review Board of the University of Hong Kong/Hospital Authority (UW 20-176).

### Definition of exposure

Patients were stratified according to the P2Y12 inhibitor (ticagrelor versus clopidogrel) prescribed upon hospital discharge. No further cross-over was allowed after the group assignment. Of note, the intravenous form of P2Y12 inhibitor was not available in our locality.

### Definition of outcomes

The primary outcome was MOF, defined as any clinical vertebral, hip and upper limb fractures (i.e. proximal humerus and wrist fractures). MOF were identified using ICD-9 CM codes validated in the electronic health record system of the Hong Kong Hospital Authority [15]. The ICD-9 CM coding system was used throughout the follow-up period [16]. Clinical vertebral fractures were identified by 805.x; upper limb fractures by 812.x, 813.x and 814.x; and hip fractures by 820.x. The secondary outcomes were the individual components of the primary outcome.

### Statistical analyses

The study cohort consisted of two comparison groups–‘ticagrelor’ and ‘clopidogrel’ groups—generated by 1:1 propensity-score-matching using a caliper of 0.2 times the standard deviation of the logit of the propensity score [17]. All analyses were performed with prespecified endpoints and statistical methods. We constructed a propensity score that predicted the likelihood of ticagrelor versus clopidogrel with variables selected a priori based on data in the published literature and biological plausibility: sex, age, ever tobacco use, diabetes, hypertension, cerebrovascular disease, chronic obstructive pulmonary disease, peripheral vascular disease, previous myocardial infarction (MI), previous heart failure, chronic kidney disease (CKD; defined as estimated glomerular filtration rate [eGFR] < 60 mL/min), anaemia before PCI (defined as haemoglobin < 13 g/dL for men and < 12 g/dL for women), obesity, previous fall event, previous MOF, inflammatory polyarthropathy, use of vitamin D or calcium supplements, use of bisphosphonates or other anti-osteoporosis medications (including raloxifene, denosumab, strontium and teriparatide), and use of proton pump inhibitors. Comparisons were performed using Chi-square tests for categorical variables and Student’s t test or Wilcoxon rank-sum tests for continuous variables. Kaplan–Meier survival curves were constructed for study groups. Cox proportional hazards regression was performed to evaluate the relationship between P2Y12 inhibitors and clinical outcomes as a time-to-first-event analysis from PCI date until death, loss to follow-up or study end date on 30 June 2022. Data management and statistical analyses were performed in Stata software, version 16 (StataCorp LP). Two-sided P values < 0.05 were considered statistically significant.

### Sensitivity analyses

The following sensitivity analyses were performed. Firstly, we included all patients before propensity score matching and repeated the primary analysis using the Cox regression model, adjusting for the same set of covariates used to construct the propensity score. Secondly, we used multiple imputation with chained equations to address missing data and repeated the primary analysis on the entire cohort using the same Cox regression model.

### Subgroup analyses

We examined any effect modification from subgroups on the relationship between P2Y12 inhibitor use and MOF by introducing interaction terms to the Cox regression model. The subgroups included age (≤ 65 vs > 65 years), sex, presence of diabetes and CKD at baseline, and history of fractures.

## Results

**Fig. 1:**
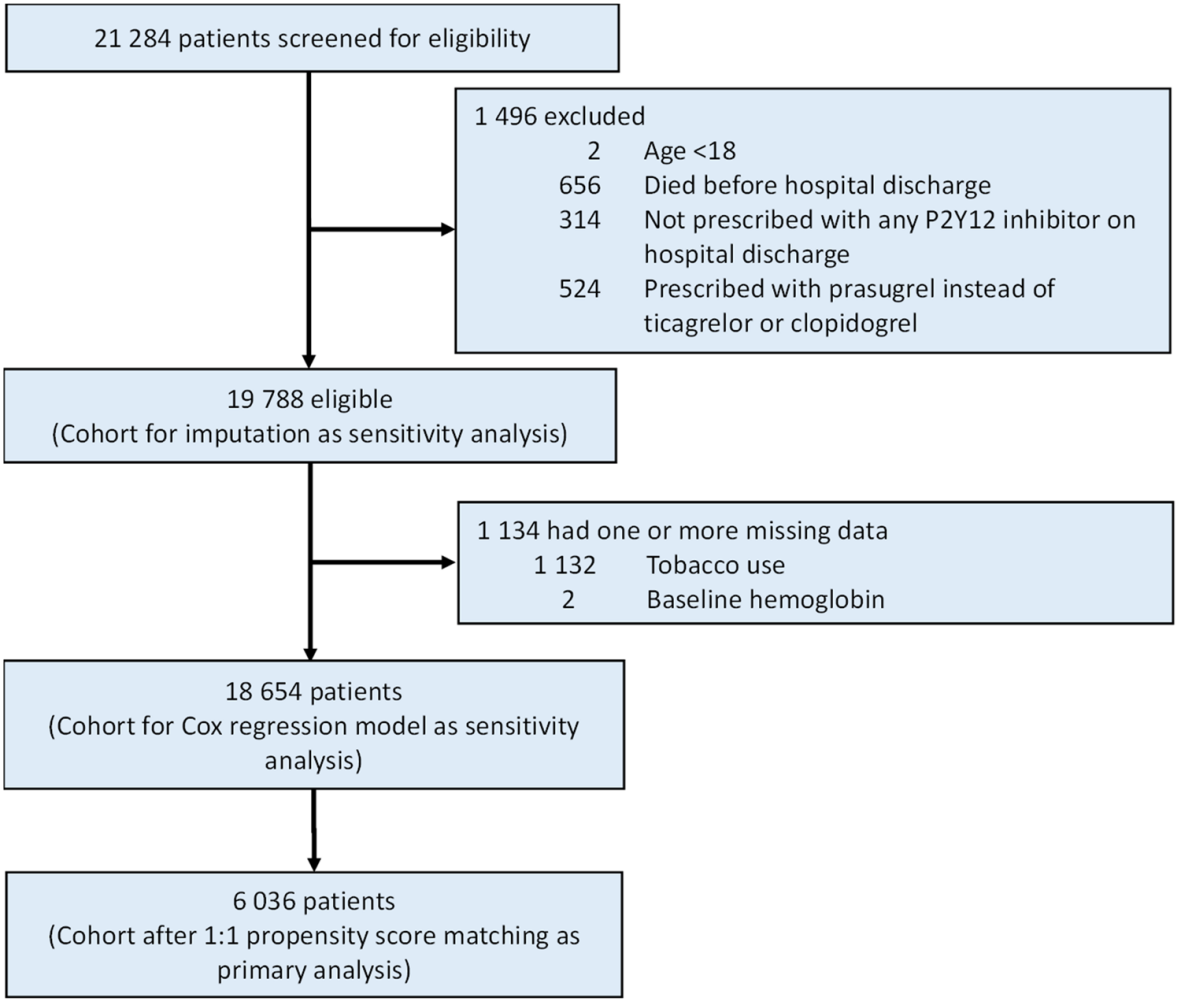
Study flow diagram

A total of 21284 patients were considered for inclusion: 1496 (7.0%) were excluded—age younger than 18 years, died before hospital discharge, not prescribed with P2Y12 inhibitor on hospital discharge, prescribed with prasugrel (instead of ticagrelor or clopidogrel) on hospital discharge. Of the remaining 19788 patients analyzed, 1134 (5.7%) were excluded from the complete case analysis due to missing values in any of the variables used in the propensity score model (Fig. 1). A total of 3018 pairs of patients prescribed either ticagrelor or clopidogrel upon hospital discharge were generated by 1:1 propensity score matching. Baseline characteristics of all patients before and after propensity score matching are shown in Supplementary Table 1 and Table 1, respectively. Before matching, the two groups had significant differences in sex, age, diabetes, hypertension, cerebrovascular disease, previous MI, previous heart failure, CKD, anemia, obesity, and baseline medications. After matching, the two groups were well balanced in the baseline characteristics with standardized differences < 0.1 for all variables (Table 1). In the matched cohort, the mean age was 61.4 ± 11.4 years, with a male predominance (only 15.9% were women). The median follow-up was 6.5 years (IQR: 5.0–7.8).

**Table 1:** Baseline characteristics of the cohort after 1:1 propensity score matching

Characteristics	Ticagrelor	Clopidogrel	P value	Standardized difference
N	3018	3018		
Female	471 (15.6%)	488 (16.2%)	0.55	0.015
Age, mean (SD)	61.4 (11.3)	61.5 (11.6)	0.66	0.011
Age > 65	1062 (35.2%)	1125 (37.3%)	0.092	0.043
Tobacco use	1549 (51.3%)	1533 (50.8%)	0.68	– 0.011
Diabetes	795 (26.3%)	810 (26.8%)	0.66	0.011
Hypertension	1480 (49.0%)	1497 (49.6%)	0.66	0.011
Cerebrovascular disease	162 (5.4%)	161 (5.3%)	0.95	– 0.001
Previous myocardial infarction	143 (4.7%)	162 (5.4%)	0.26	0.029
Previous heart failure	100 (3.3%)	110 (3.6%)	0.48	0.018
eGFR in ml/min, mean (SD)	81.4 (23.0)	79.6 (22.4)	0.003	– 0.076
eGFR below 60 ml/min	492 (16.3%)	496 (16.4%)	0.89	0.004
Hemoglobin in g/dL, mean (SD)	13.8 (1.7)	13.6 (1.7)	< 0.001	– 0.089
Anemia*	713 (23.6%)	701 (23.2%)	0.72	– 0.009
Obesity	37 (1.2%)	46 (1.5%)	0.32	0.026
Previous fall event	260 (8.6%)	296 (9.8%)	0.11	0.041
Previous major osteoporotic fracture	122 (4.0%)	142 (4.7%)	0.21	0.032
Polyarthropathy	18 (0.6%)	16 (0.5%)	0.73	– 0.009
Medication received				0.002
Calcium/vitamin D supplements	75 (2.5%)	76 (2.5%)	0.93	0.015
Calcium supplements	69 (2.3%)	76 (2.5%)	0.56	– 0.015
Vitamin D supplements	26 (0.9%)	19 (0.6%)	0.29	– 0.027
Anti-osteoporosis agents	7 (0.2%)	7 (0.2%)	1.00	< 0.001
Bisphosphonates	5 (0.2%)	5 (0.2%)	1.00	< 0.001
Proton pump inhibitors	2466 (81.7%)	2493 (82.6%)	0.36	0.023

*SD* standard deviation, *eGFR* estimated glomerular filtration rate

*Anemia: Hemoglobin < 13 g/dL for men, < 12 g/dL for women

### Primary outcomes

**Fig. 2:**
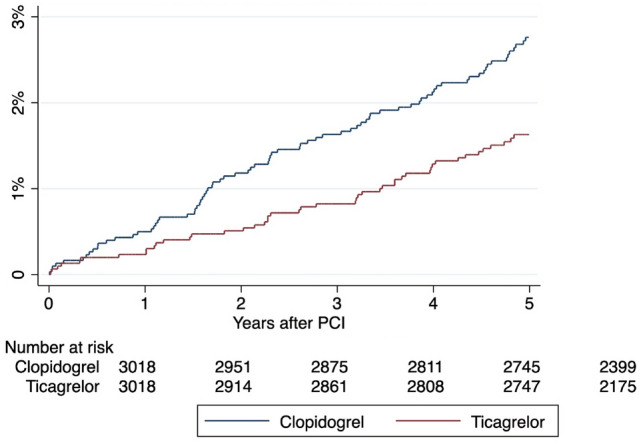
Estimated probabilities of major osteoporotic fracture stratified by P2Y12 inhibitors

MOF occurred in 59 (2.0%) patients on ticagrelor and 119 (3.9%) patients on clopidogrel, corresponding to an annualized risk of 0.34% and 0.56%, respectively. The hazard ratio (HR) of MOF associated with ticagrelor use was 0.60 (95% CI, 0.44–0.83; P = 0.002) with reference to clopidogrel use (Table 2 and Fig. 2).

**Table 2:** Annualized risks and hazard ratios of primary and secondary outcomes in the ticagrelor and clopidogrel groups

Outcomes	Ticagrelor (95% CI)	Clopidogrel (95% CI)	Hazard ratio (95% CI)	P value
Primary outcome				
Any major osteoporotic fracture	0.34% (0.26–0.44%)	0.56% (0.47–0.68%)	0.60 (0.44–0.83)	0.002
Secondary outcomes				
Vertebral fracture	0.06% (0.03–0.11%)	0.10% (0.06–0.15%)	0.61 (0.29–1.27)	0.19
Hip fracture	0.14% (0.10–0.21%)	0.24% (0.19–0.32%)	0.65 (0.40–1.06)	0.087
Upper limb fracture	0.15% (0.11–0.23%)	0.28% (0.22–0.36%)	0.53 (0.34–0.85)	0.008

### Secondary outcomes

The HR of fractures at individual sites was consistent with the main analysis. While the risk of upper limb fracture was lower in the ticagrelor group reaching statistical significance (HR, 0.53; 95% CI, 0.34–0.85, P = 0.008), the HR of clinical vertebral fractures (HR, 0.61; 95% CI, 0.29–1.27; P = 0.19) and hip fractures (HR, 0.65; 95% CI, 0.40–1.06; P = 0.087) suggested lower fracture risks in the ticagrelor group although not reaching statistical significance, possibly because of the lower event rates (Table 2 and Supplementary Figs. 1–3).

### Sensitivity analyses

Both sensitivity analyses were consistent with the results of the main analysis. In the first sensitivity analysis, we analyzed the outcomes of all 18,654 patients with complete information before propensity score matching. After adjusting for potential confounders using the Cox regression model, the risk of any MOF was lower in the ticagrelor group (HR, 0.72; 95% CI, 0.55–0.94; P = 0.017). Similar results were obtained in the analysis of secondary outcomes: HR of fractures at individual fracture sites associated with ticagrelor use—clinical vertebral fracture (HR, 0.78; 95% CI, 0.42–1.46; P = 0.44), upper limb fracture (HR, 0.78; 95% CI, 0.52–1.18; P = 0.25) and hip fracture (HR, 0.64; 95% CI, 0.43–0.97; P = 0.033). In the second sensitivity analysis, we analyzed the outcomes of all 19,788 patients using multiple imputation with chained equations to address missing data. After adjusting for potential confounders using the Cox regression model, the HR of MOF associated with ticagrelor use was 0.73 (95% CI, 0.57–0.95; P = 0.019) with reference to clopidogrel use.

### Subgroup analyses

**Fig. 3:**
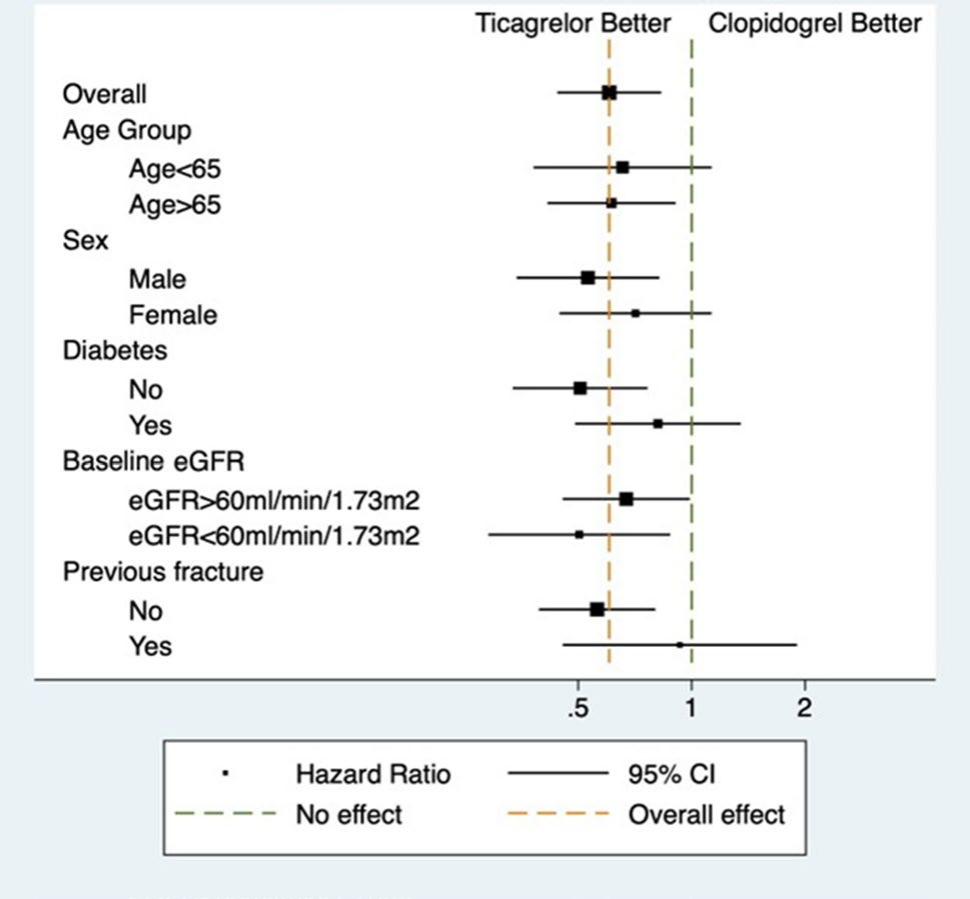
Forest plot showing subgroup analyses of the risks of major osteoporotic fractures

In the subgroup analyses, we did not observe significant interaction in the relationship between P2Y12 inhibitors and MOF across different subgroups—age, sex, presence of diabetes, presence of CKD and history of fractures (Table 3 and Fig. 3).

**Table 3:** Subgroup analysis examining differential effects of P2Y12 inhibitors on the risk of major osteoporotic fractures

Subgroup	Hazard ratio	95% Confidence interval		P value for interaction
Age group				0.80
≤ 65 years	0.66	0.38	1.13	
> 65 years	0.61	0.41	0.91	
Sex				0.34
Male	0.53	0.34	0.82	
Female	0.71	0.45	1.13	
Diabetes				0.18
No	0.51	0.33	0.76	
Yes	0.81	0.49	1.35	
Baseline eGFR				0.47
≥ 60 mL/min	0.67	0.45	0.99	
< 60 mL/min	0.50	0.29	0.88	
Previous fracture				0.14
No	0.56	0.39	0.80	
Yes	0.93	0.45	1.90

## Discussion

This is the first study in the literature addressing the impact of the choice of P2Y12 inhibitor in the DAPT regimen on the risk of MOF. Among patients undergoing PCI for ACS, ticagrelor use was associated with reduced risks of MOF compared with clopidogrel. From the perspective of bone health, ticagrelor may be the preferred agent in the DAPT regimen.

Ticagrelor, a potent P2Y12 inhibitor, has superior efficacy over clopidogrel in treating CAD [18, 19]. In line with the results of the randomized controlled trials, our group has previously reported the superiority of potent P2Y12 inhibitors (predominantly ticagrelor) over clopidogrel in our current post-PCI cohort with a lower hazard of MI (including but not exclusively reinfarction) after PCI in the potent P2Y12 inhibitor group (adjusted HR 0.71; 95% CI 0.59–0.87; P < 0.001) [14]. Therefore, further evaluation of the fracture risk among patients receiving clopidogrel versus ticagrelor in the post-PCI cohort is essential. Patients with ACS undergoing PCI are at an increased risk of fracture per se. Previous observational studies have demonstrated an increased risk of osteoporosis and vertebral fracture following coronary events [3, 18]. Furthermore, given the role of aspirin in DAPT, proton pump inhibitors (PPI) are often co-prescribed with aspirin to reduce the risk of peptic ulcer disease [20]. PPI is a known risk factor for osteoporosis, increasing the risk of osteoporotic fractures [21, 22]. In our cohort, over 80% of the individuals were put on PPI. Overall, we observed an annualized fracture risk of 0.3–0.6%. Given the male preponderance and the relatively young age of the cohort (mean age of around 60 years), this level of fracture risk required attention.

Focusing on patients with ACS treated with first-ever PCI, our study demonstrated a higher risk of MOF with clopidogrel than ticagrelor, favouring using the latter agent in the post-PCI setting. While the association between clopidogrel use and fracture risk has been reported in prior studies [10], there is a lack of relevant studies on potent P2Y12 inhibitors such as ticagrelor. The nationwide cohort study by Jorgensen et al. showed that treatment with clopidogrel was associated with an increased risk of overall fractures and osteoporotic fractures, especially in patients with treatment duration longer than one year [10]. A dose-dependent relationship was also observed, where individuals on higher doses of clopidogrel had an increased risk of fractures [10]. Even though the latest study by Jorgensen et al. revealed no significant association between clopidogrel treatment and fracture risk in patients with stroke and transient ischemic attack [23], that might be explained by the fact that such patients were immobilized in bed for prolonged periods, which reduced the risk of falls and subsequent fractures [23].

The difference in the fracture risk between ticagrelor and clopidogrel users could be understood from the respective effects of the P2Y12 inhibitors on the bone in pre-clinical studies. Syberg et al. showed that clopidogrel delays osteoblast growth and reduces cell viability [24]. The resulting decrease in mature osteoblasts causes a decline in alkaline phosphatase activity and collagen production, inhibiting new bone formation. Furthermore, clopidogrel induces the precursor cells to preferentially differentiate into the adipocytic lineage instead of the osteoblastic lineage, creating a negative bone balance [24]. While P2Y12 receptors are expressed in both osteoblasts and osteoclasts, the effect of clopidogrel on the reduction of osteoclast activity and bone resorption is smaller in magnitude than that on the reduction of osteoblast differentiation and bone formation [24]. Hence, Syberg et al. postulated that prolonged exposure to clopidogrel could negatively impact bone health. On the other hand, the lower fracture risk observed with ticagrelor use could be ascribed to its ability to enhance bone formation via an adenosine-dependent mechanism. Mediero et al. demonstrated that in vivo, direct or indirect stimulation of adenosine A_2A_ receptor (A2AR) by specific agonists or by enhancing endogenous adenosine levels enhances new bone formation and regeneration [25]. Meanwhile, ticagrelor regulates osteoblast and osteoclast function by blocking adenosine uptake, increasing extracellular adenosine levels, thereby stimulating A2AR [12]. A2AR activation modulates the expression of semaphorins 4D (Sema4D) and 3A (Sema3A), which are important regulators of bone homeostasis [26]. Specifically, A2AR stimulation reduces the release of Sema4D by osteoclasts, inhibits Sema4D-mediated osteoclast activation and increases the secretion of Sema3A by osteoblasts [26]. This helps promote osteoblast differentiation and inhibit osteoclast activity, consequently increasing new bone formation.

Secondary analyses revealed consistently lower HR of fractures over vertebrae, hip and upper limbs associated with ticagrelor use. However, the HR of fractures over vertebrae and hip did not reach statistical significance, which may be explained by the lower number of events at these two sites. In the study by Jorgensen et al., fracture risk over different sites (any fractures, hip, forearm and spine) was consistently higher in the clopidogrel-treated group compared to those not treated with clopidogrel [10]. This suggests that the differential effects of clopidogrel versus ticagrelor on the bone are probably systemic. Furthermore, subgroup analyses showed that the observed protective effects of ticagrelor over clopidogrel on fracture risk were consistent across various subgroups, denoting individuals with different fracture risk profiles—age, sex, presence of diabetes and CKD at baseline, and prior history of fractures. Our results suggested the favorable effects of ticagrelor over clopidogrel on bone health across patients with different baseline fracture risks.

There are several limitations in the present study. First, although we applied propensity score matching for various baseline characteristics, including the fracture risk profile of the clopidogrel and ticagrelor groups, the issue of unmeasured confounding and bias could not be entirely excluded given the observational nature of this cohort study. Secondly, bone mineral density measurement and bone turnover markers were not available. Nonetheless, bone mineral density was not the typical determinant of eligibility for various P2Y12 inhibitors. In addition, fracture events were the more important outcomes from patients’ perspective. Thirdly, treatment adherence could not be ascertained like all large-scale pharmacoepidemiologic studies using electronic health databases. Fourthly, as vertebral fractures were not systematically assessed by quantitative vertebral morphometry, we could only identify clinical, but not morphometric, vertebral fractures. Vertebral fractures are frequently asymptomatic [27], so morphometric vertebral fractures would go undetected. This could lead to an underestimation of the number of events of vertebral fractures. Finally, this study was performed among Chinese patients. The results may not be generalizable to patients of other ethnicities.

## Conclusion

In this cohort of Chinese patients who underwent PCI for ACS, ticagrelor was associated with a lower risk of MOF than clopidogrel use. Our results support the use of ticagrelor in the DAPT regimen from the perspective of bone health.

### Supplementary Information

Below is the link to the electronic supplementary material.

Supplementary file1 (DOCX 433 KB)

## Data Availability

The datasets generated during and/or analysed during the current study are available from the corresponding author on reasonable request.

## References

[1] Rachner TD, Khosla S, Hofbauer LC (2011). Osteoporosis: now and the future. Lancet.

[2] Johnell O, Kanis JA (2006). An estimate of the worldwide prevalence and disability associated with osteoporotic fractures. Osteoporos Int.

[3] Syu DK, Hsu SH, Yeh PC, Kuo YF, Huang YC, Jiang CC (2022). The association between coronary artery disease and osteoporosis: a population-based longitudinal study in Taiwan. Arch Osteoporos.

[4] Laroche M, Pécourneau V, Blain H, Breuil V, Chapurlat R, Cortet B (2017). Osteoporosis and ischemic cardiovascular disease. Joint Bone Spine.

[5] Cuisset T, Verheugt FWA, Mauri L (2017). Update on antithrombotic therapy after percutaneous coronary revascularisation. Lancet.

[6] Valgimigli M, Bueno H, Byrne RA, Collet JP, Costa F, Jeppsson A (2018). 2017 ESC focused update on dual anti-platelet therapy in coronary artery disease developed in collaboration with EACTS: the task force for dual anti-platelet therapy in coronary artery disease of the European society of cardiology (ESC) and of the European association for cardio-thoracic surgery (EACTS). Eur Heart J.

[7] Wiviott SD, Braunwald E, McCabe CH, Montalescot G, Ruzyllo W, Gottlieb S (2007). Prasugrel versus clopidogrel in patients with acute coronary syndromes. N Engl J Med.

[8] Wallentin L, Becker RC, Budaj A, Cannon CP, Emanuelsson H, Held C (2009). Ticagrelor versus clopidogrel in patients with acute coronary syndromes. N Engl J Med.

[9] Vestergaard P, Steinberg TH, Schwarz P, Jørgensen NR (2012). Use of the oral platelet inhibitors dipyridamole and acetylsalicylic acid is associated with increased risk of fracture. Int J Cardiol.

[10] Jørgensen NR, Grove EL, Schwarz P, Vestergaard P (2012). Clopidogrel and the risk of osteoporotic fractures: a nationwide cohort study. J Intern Med.

[11] Buckley KA, Golding SL, Rice JM, Dillon JP, Gallagher JA (2003). Release and interconversion of P2 receptor agonists by human osteoblast-like cells. Faseb J.

[12] Mediero A, Wilder T, Reddy VS, Cheng Q, Tovar N, Coelho PG (2016). Ticagrelor regulates osteoblast and osteoclast function and promotes bone formation in vivo via an adenosine-dependent mechanism. Faseb J.

[13] von der Recke P, Hansen MA, Hassager C (1999). The association between low bone mass at the menopause and cardiovascular mortality. Am J Med.

[14] Ng AK, Ng PY, Ip A, Lau KK, Siu CW (2022). Risk of ischaemic and haemorrhagic stroke in Chinese undergoing percutaneous coronary intervention treated with potent P2Y12 inhibitor versus clopidogrel. Stroke Vasc Neurol.

[15] Lui DTW, Ho Man Tang E, Au ICH, Wu T, Lee CH, Wong CK (2022). Evaluation of fracture risk among patients with type 2 diabetes and nonvalvular atrial fibrillation receiving different oral anticoagulants. Diabetes Care.

[16] Xiong X, Lui DTW, Chung MSH, Au ICH, Lai FTT, Wan EYF (2023). Incidence of diabetes following COVID-19 vaccination and SARS-CoV-2 infection in Hong Kong: a population-based cohort study. PLoS Med.

[17] Austin PC (2011). Optimal caliper widths for propensity-score matching when estimating differences in means and differences in proportions in observational studies. Pharm Stat.

[18] Silva HC, Pinheiro MM, Genaro PS, Castro CH, Monteiro CM, Fonseca FA (2013). Higher prevalence of morphometric vertebral fractures in patients with recent coronary events independently of BMD measurements. Bone.

[19] Sharma R, Kumar P, Prashanth SP, Belagali Y (2020). Dual anti-platelet therapy in coronary artery disease. Cardiol Ther.

[20] Han YY, Li ZX, Duan R (2021). Efficacy and safety of proton pump inhibitors combined with clopidogrel in patients undergoing percutaneous coronary intervention: a meta-analysis. Rev Cardiovasc Med.

[21] Ngamruengphong S, Leontiadis GI, Radhi S, Dentino A, Nugent K (2011). Proton pump inhibitors and risk of fracture: a systematic review and meta-analysis of observational studies. Am J Gastroenterol.

[22] Zhou B, Huang Y, Li H, Sun W, Liu J (2016). Proton-pump inhibitors and risk of fractures: an update meta-analysis. Osteoporos Int.

[23] Jørgensen NR, Schwarz P, Iversen HK, Vestergaard P (2017). P2Y12 receptor antagonist, clopidogrel, does not contribute to risk of osteoporotic fractures in stroke patients. Front Pharmacol.

[24] Syberg S, Brandao-Burch A, Patel JJ, Hajjawi M, Arnett TR, Schwarz P (2012). Clopidogrel (Plavix), a P2Y12 receptor antagonist, inhibits bone cell function in vitro and decreases trabecular bone in vivo. J Bone Miner Res.

[25] Mediero A, Wilder T, Perez-Aso M, Cronstein BN (2015). Direct or indirect stimulation of adenosine A2A receptors enhances bone regeneration as well as bone morphogenetic protein-2. Faseb j.

[26] Mediero A, Wilder T, Shah L, Cronstein BN (2018). Adenosine A(2A) receptor (A2AR) stimulation modulates expression of semaphorins 4D and 3A, regulators of bone homeostasis. Faseb J.

[27] Schousboe JT (2016). Epidemiology of vertebral fractures. J Clin Densitom.

