# Supplementary material for: Ticagrelor was associated with lower fracture risk than clopidogrel in the dual anti-platelet regimen among patients with acute coronary syndrome treated with percutaneous coronary intervention
Source: J Endocrinol Invest. 2023 Sep 30;47(4):895–902. doi: 10.1007/s40618-023-02205-1 (PMC10965638; doi:10.1007/s40618-023-02205-1)

**Supplementary Table 1.** Baseline characteristics of the cohort before propensity score matching

| Characteristics | Ticagrelor | Clopidogrel | P value | Standardized difference |
| --- | --- | --- | --- | --- |
| N | 3199 | 16589 |  |  |
| Female | 486 (15.2%) | 3913 (23.6%) | <0.001 | 0.214 |
| Age, mean (SD) | 61.3 (11.3) | 65.2 (11.6) | <0.001 | 0.340 |
| Age > 65 | 1125 (35.2%) | 8414 (50.7%) | <0.001 | 0.318 |
| Tobacco use | 1551 (51.4%) | 7440 (47.6%) | <0.001 | -0.076 |
| Diabetes | 825 (25.8%) | 5939 (35.8%) | <0.001 | 0.218 |
| Hypertension | 1544 (48.3%) | 10753 (64.8%) | <0.001 | 0.339 |
| Cerebrovascular disease | 172 (5.4%) | 1724 (10.4%) | <0.001 | 0.187 |
| Previous myocardial infarction | 152 (4.8%) | 2358 (14.2%) | <0.001 | 0.327 |
| Previous heart failure | 107 (3.3%) | 1436 (8.7%) | <0.001 | 0.225 |
| eGFR in mL/min, mean (SD) | 81.3 (23.0) | 74.5 (24.8) | <0.001 | -0.281 |
| eGFR <60 mL/min | 529 (16.5%) | 4039 (24.3%) | <0.001 | 0.195 |
| Hemoglobin in g/dL, mean (SD) | 13.8 (1.7) | 13.2 (1.8) | <0.001 | -0.356 |
| Anemia* | 755 (23.6%) | 5714 (34.4%) | <0.001 | 0.240 |
| Obesity | 38 (1.2%) | 217 (1.3%) | 0.58 | 0.108 |
| Previous fall event | 271 (8.5%) | 1535 (9.3%) | 0.16 | 0.027 |
| Previous major osteoporotic fracture | 128 (4.0%) | 752 (4.5%) | 0.18 | 0.026 |
| Polyarthropathy | 19 (0.6%) | 115 (0.7%) | 0.53 | 0.012 |
| Medications received |  |  |  |  |
| Calcium/vitamin D supplements | 79 (2.5%) | 766 (4.6%) | <0.001 | 0.116 |
| Calcium supplements | 73 (2.3%) | 745 (4.5%) | <0.001 | 0.122 |
| Vitamin D supplements | 27 (0.8%) | 142 (0.9%) | 0.95 | 0.001 |
| Anti-osteoporosis agents | 8 (0.3%) | 58 (0.3%) | 0.37 | -0.474 |
| Bisphosphonates | 6 (0.2%) | 50 (0.3%) | 0.27 | 0.018 |
| Proton pump inhibitors | 2609 (81.6%) | 10058 (60.6%) | <0.001 | 0.023 |

Abbreviations: SD, standard deviation; eGFR, estimated glomerular filtration rate.

*Anemia: Hemoglobin <13g/dL for men, <12g/dL for women.

**Supplementary Figure 1.** Estimated probabilities of clinical vertebral fractures stratified by P2Y12 inhibitor


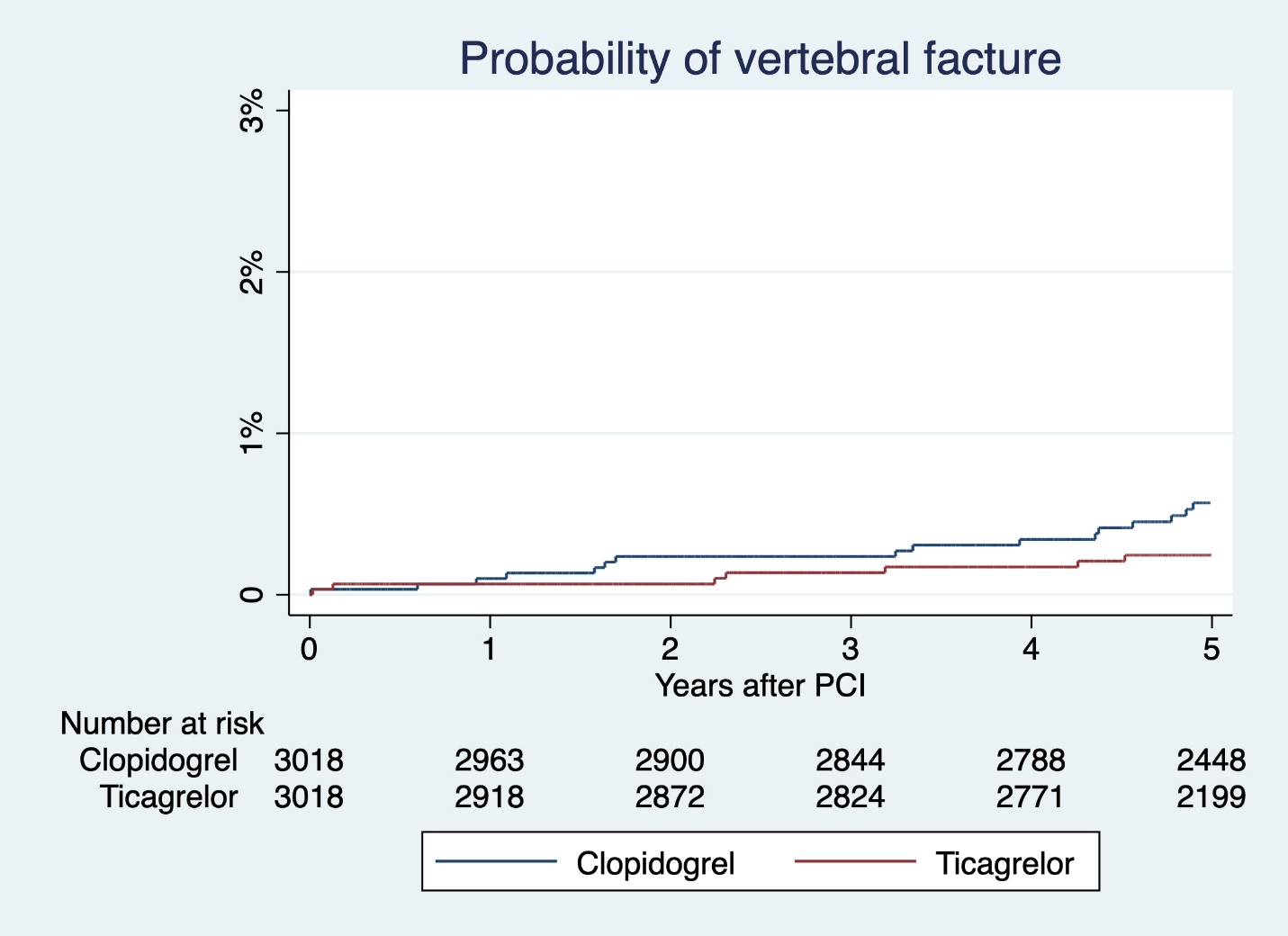


**Supplementary Figure 2.** Estimated probabilities of hip fractures stratified by P2Y12 inhibitor


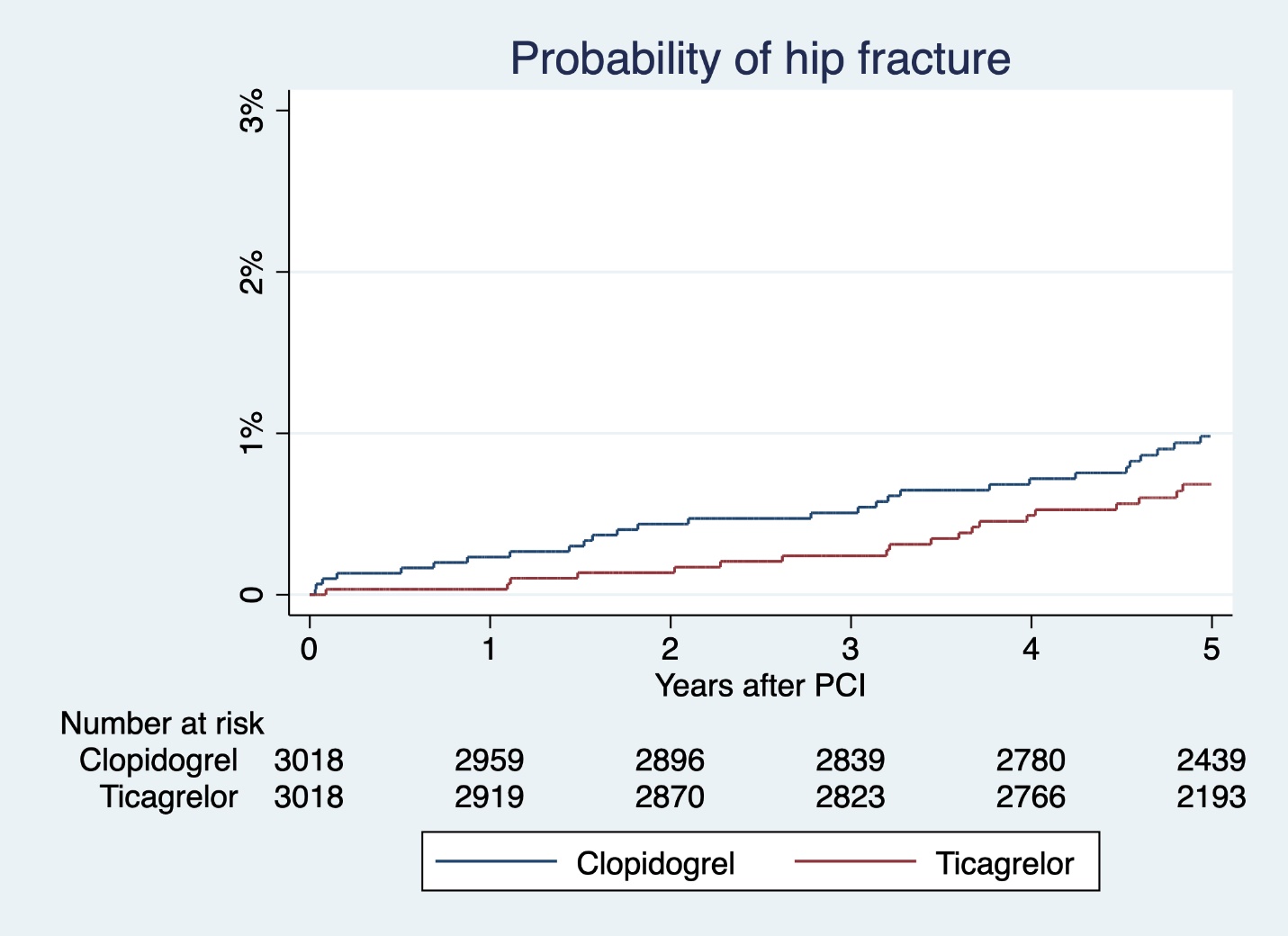


**Supplementary Figure 3.** Estimated probabilities of upper limb fractures stratified by P2Y12 inhibitor


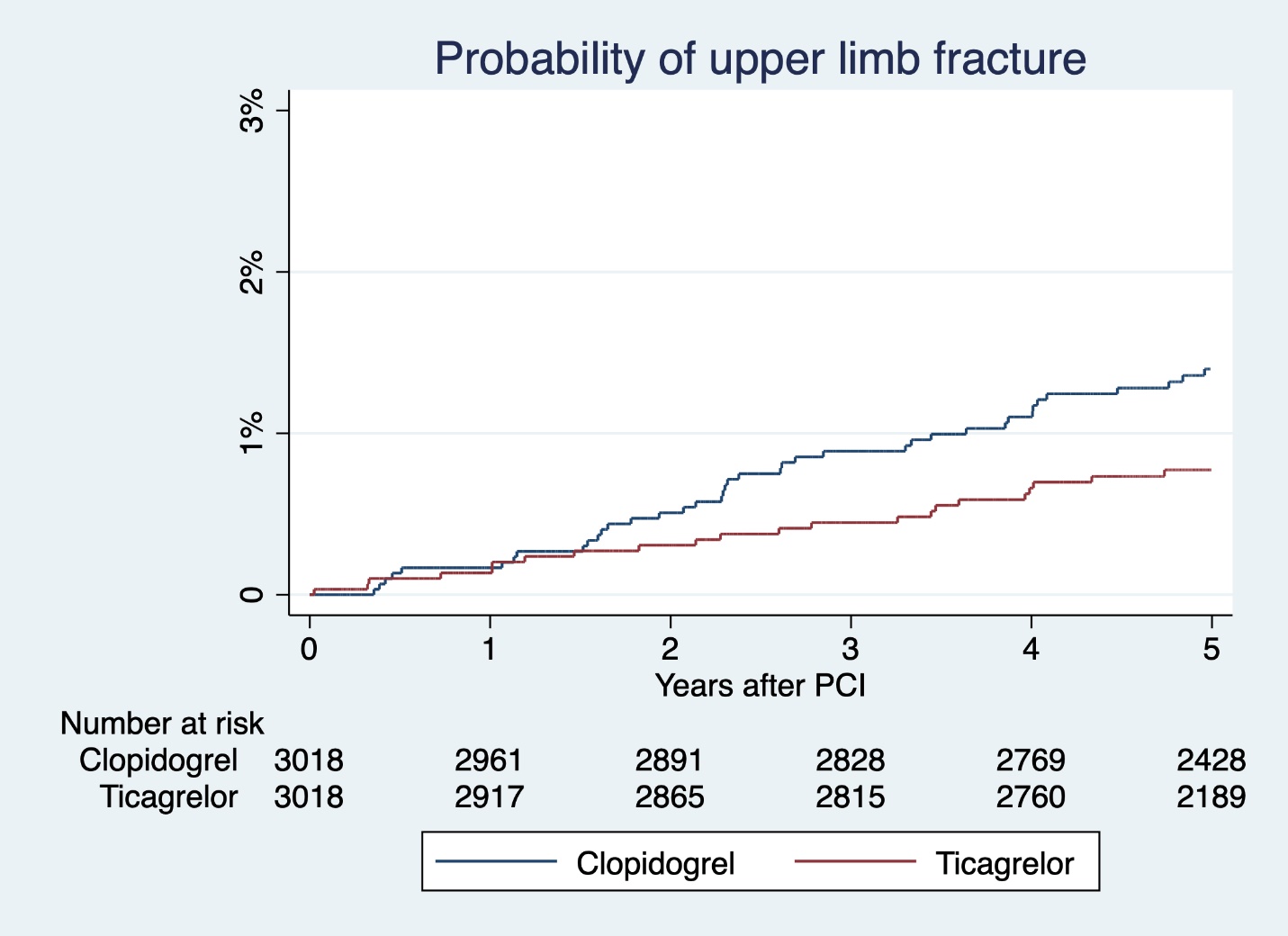

Supplement: Supplementary file 1 — Supplementary file1 (DOCX 433 KB) [file 40618_2023_2205_MOESM1_ESM.docx]
